# Racial and Socioeconomic Disparities in Survival Among Patients with Metastatic Prostate Cancer: A SEER Population-Based Study

**DOI:** 10.3390/cancers18101496

**Published:** 2026-05-07

**Authors:** Onyekachi Anya, Ogbonna Chikere, Progress Asoluka, Helen Oletu

**Affiliations:** 1 Department of Internal Medicine, Legacy Salmon Creek Medical Center, Vancouver, WA 98686, USA; 2 Department of Internal Medicine, North Knoxville Medical Center, Knoxville, TN 37849, USA

**Keywords:** prostate cancer, racial disparities, socioeconomic status, cancer-specific survival, SEER database, metastatic prostate cancer

## Abstract

**Background**: Prostate cancer remains a major cause of cancer morbidity and mortality among men in the United States. Differences in diagnosis and survival across racial and socioeconomic groups continue to raise concern in clinical and public health research. Population-based datasets provide an opportunity to examine patterns of advanced disease and survival outcomes across diverse demographic groups. **Objective**: This study evaluated racial and socioeconomic disparities in cancer-specific survival among patients with metastatic prostate cancer using a national population-based dataset. **Methods**: A retrospective population-based study was conducted using data from the Surveillance, Epidemiology, and End Results (SEER) program. Patients diagnosed with malignant prostate cancer between 2004 and 2020 with distant stage disease were included. The final analytic sample consisted of 54,062 patients. Variables included race and ethnicity, age group, metastatic sites at diagnosis, treatment variables, and median household income. Descriptive analyses compared characteristics by cancer-specific death using chi-square tests for categorical variables and *t* tests for continuous variables. Survival patterns were examined using Kaplan–Meier methods and log-rank tests. Multivariable Cox proportional hazards model was used to estimate adjusted hazard ratios for factors associated with cancer-specific mortality. **Results**: Cancer-specific mortality differed across racial and socioeconomic groups. Higher mortality was observed among non-Hispanic Black patients (aHR = 1.15, 95% CI: 1.00 to 1.31, *p* = 0.046) and non-Hispanic American Indian or Alaska Native patients (aHR = 1.15, 95% CI: 1.10 to 1.20, *p* < 0.001) compared with non-Hispanic White patients, while Hispanic and non-Hispanic Asian or Pacific Islander patients showed lower mortality risk. Older age groups demonstrated higher mortality. Liver, lung, and brain metastases were associated with increased risk of prostate cancer death. Patients in higher income groups showed lower mortality compared with patients in lower income groups (aHR = 0.83, 95% CI: 0.80 to 0.87, *p* < 0.001). **Conclusions**: This study highlights persistent racial and socioeconomic differences in cancer-specific survival among patients with advanced prostate cancer in the United States. These findings support continued efforts to address disparities in early detection, access to care, and treatment pathways. Future research should further explore clinical and structural factors that influence survival differences across population groups.

## Simple Summary

Prostate cancer is a leading cause of cancer-related mortality among men in the United States, with survival outcomes influenced by racial and socioeconomic factors. This study used data from the SEER program to examine disparities in cancer-specific survival among 54,062 patients with metastatic prostate cancer diagnosed between 2004 and 2020. We found that non-Hispanic Black and non-Hispanic American Indian or Alaska Native patients had higher mortality compared with non-Hispanic White patients, while Hispanic and non-Hispanic Asian or Pacific Islander patients had lower risk. Patients living in lower income areas also experienced worse outcomes. Metastases to the liver and brain were associated with particularly poor survival. These results emphasize the need to address structural and social determinants of health, improve access to early detection, and ensure equitable treatment to reduce disparities in prostate cancer outcomes.

## 1. Introduction

Prostate cancer is the most frequently diagnosed malignancy among men in the United States and remains a major contributor to cancer-related mortality [1]. Advances in screening, early detection, and treatment have improved outcomes over recent decades; however, a substantial proportion of patients continue to present with advanced or metastatic disease, which is associated with significantly poor survival outcomes. Early detection through prostate-specific antigen (PSA) screening has been shown to reduce prostate cancer-specific mortality and the incidence of metastatic disease at diagnosis [2]. Furthermore, active surveillance has emerged as an important management strategy for men with low-risk screen-detected prostate cancer, helping to mitigate the harms of overtreatment while maintaining oncologic safety [3]. Despite these advances, prostate cancer continues to represent an important public health challenge due to persistent differences in disease burden and outcomes across population groups.

Disparities in prostate cancer outcomes extend beyond detection and are particularly pronounced in advanced disease. Metastatic prostate cancer disproportionately affects certain racial groups, with differences in metastatic burden, treatment access, and survival outcomes documented across populations. Systematic reviews have identified persistent racial disparities in prostate cancer incidence, treatment patterns, and mortality, with Black men consistently experiencing worse outcomes compared with other racial groups [4,5,6].

Racial disparities in prostate cancer outcomes are well documented in the United States. Numerous studies have demonstrated that Black men experience higher incidence rates, are more likely to present with aggressive disease, and have higher prostate cancer-specific mortality compared with other racial groups [7,8,9]. These differences are believed to arise from a complex interaction between biological variation, environmental exposures, structural determinants of health, and differences in access to screening, diagnosis, and treatment [10]. Social determinants of health, including income, education level, insurance coverage, and access to preventive health services, play an important role in influencing cancer outcomes across populations [11,12].

Socioeconomic status has also been identified as an important factor affecting prostate cancer detection and survival. Individuals with lower socioeconomic status often face barriers to early diagnosis and optimal treatment, including reduced access to screening services, delays in specialist referral, financial limitations, and limited access to high-quality cancer care [13,14,15]. These barriers may contribute to later stages at diagnosis and poorer survival outcomes among disadvantaged populations [16]. Previous research has documented disparities in prostate cancer incidence, screening patterns, and treatment utilization across racial and socioeconomic groups [4,5,9,17]. However, many prior studies have focused primarily on localized disease or treatment patterns, and fewer investigations have examined survival differences among patients presenting with advanced or metastatic prostate cancer at diagnosis [18]. Additionally, some existing studies are limited to single institutions or regional cohorts, which may not fully capture national trends in disparities [19]. Population-based cancer registries provide an important opportunity to evaluate disparities in cancer outcomes at a national level. The Surveillance, Epidemiology, and End Results (SEER) program is a comprehensive cancer registry that collects detailed information on cancer incidence, patient demographics, tumor characteristics, treatment, and survival across multiple geographic regions in the United States [20]. The large sample size and population-based design of SEER make it a valuable resource for examining demographic and socioeconomic differences in cancer outcomes. Prior population-based analyses have demonstrated significant variation in prostate cancer mortality among Hispanic/Latino subgroups, underscoring the importance of disaggregated analyses when evaluating outcomes across diverse ethnic populations [21].

The objective of this study was to evaluate racial and socioeconomic disparities in cancer-specific survival among patients diagnosed with metastatic prostate cancer using data from the SEER program. We hypothesized that patients from racial minority groups and those residing in lower socioeconomic areas would experience higher prostate cancer-specific mortality compared with non-Hispanic White patients and individuals from higher income areas.

## 2. Methodology

### 2.1. Study Design and Data Source

This study used a retrospective population-based design to examine disparities in stage at diagnosis and cancer-specific survival among patients with advanced prostate cancer in the United States. Data were obtained from the Surveillance, Epidemiology, and End Results (SEER) program, a population-based cancer registry system supported by the National Cancer Institute (NCI). SEER collects and publishes cancer incidence, treatment, and survival data from multiple population-based registries that together represent approximately 28% of the U.S. population. The database includes detailed demographic, tumor, and survival information and is widely used for epidemiological and outcomes research. SEER data are publicly available and can be accessed by researchers upon registration and approval through the NCI [17].

### 2.2. Study Population

The study population included patients diagnosed with primary prostate cancer identified using the International Classification of Diseases for Oncology site code C61.9. Only malignant tumors were included. Patients diagnosed between 2004 and 2020 were selected to allow sufficient follow up and consistent reporting of clinical variables. Although the SEER database includes patients across all age groups, this study was restricted to individuals aged 40 years and older, as prostate cancer predominantly affects this population and is rare in younger individuals. The initial cohort extracted from the SEER database included 54,118 patients. The analysis focused on patients with advanced disease defined as distant stage at diagnosis based on the SEER combined summary stage classification. After applying these criteria and excluding records with incomplete survival information, the final analytic sample included 54,062 patients with advanced prostate cancer.

### 2.3. Variables and Measures

The primary outcome was cancer-specific death. This outcome was derived from the SEER cause-specific death classification variable and categorized as death attributable to prostate cancer or alive or dead from another cause. Survival time was measured in months from the date of diagnosis to the date of death or last follow up. The main explanatory variables included race and ethnicity, age group at diagnosis, metastatic sites at diagnosis, treatment variables, and median household income. Race and ethnicity were categorized as non-Hispanic White, non-Hispanic Black, non-Hispanic Asian or Pacific Islander, non-Hispanic American Indian or Alaska Native, Hispanic, and non-Hispanic unknown. Age was grouped into ten-year categories beginning with 40–44 years and extending to 90 years and older. Metastatic sites at diagnosis included bone metastasis, liver metastasis, lung metastasis, and brain metastasis. Each metastatic variable was categorized as present or absent. Treatment variables included receipt of radiation therapy and chemotherapy. Socioeconomic status was represented by median household income grouped into four categories: low income (<$60 k), lower-middle income ($60–79 k), upper-middle income ($80–99 k), and high income (≥$100 k). Descriptive statistics and group comparisons were performed as described in the Section 2.5.

### 2.4. Missing Data

The proportion of missing data was minimal for most variables included in the analysis. Radiotherapy had a small proportion of missing observations, representing 2.9 percent of the dataset. These observations were excluded from analyses involving radiotherapy to maintain consistency in statistical comparisons.

### 2.5. Statistical Analysis

Descriptive statistics were used to summarize demographic, clinical, and socioeconomic characteristics of the study population. Categorical variables were reported as counts and percentages, while continuous variables were summarized using mean and standard deviation. Group comparisons by cancer-specific mortality were conducted using chi-square tests for categorical variables and independent sample *t* tests for continuous variables. Survival analyses were performed using Kaplan–Meier methods to estimate cancer-specific survival across race and income groups. Differences between survival curves were evaluated using the log-rank test. Multivariable Cox proportional hazards regression model was used to estimate adjusted hazard ratios and corresponding confidence intervals for factors associated with cancer-specific mortality. The model included demographic characteristics, metastatic sites, treatment variables, and median household income categories. All statistical analyses were conducted using Stata version 18.

### 2.6. Ethical Considerations

This study used publicly available, deidentified data from the SEER program [17]. Because the dataset does not contain identifiable personal information, the analysis did not involve direct contact with human subjects. Research using SEER data is considered exempt from institutional review board oversight according to federal guidelines for studies involving deidentified public datasets.

## 3. Results

Table 1 below presents the baseline characteristics of patients with advanced prostate cancer according to cancer-specific death status.

This study included 54,062 patients with advanced prostate cancer. Among these patients, 21,544 patients were classified as alive or died from other causes, while 32,518 patients died from prostate cancer.

Age distribution differed significantly between the outcome groups (χ^2^ = 241.34, *p* < 0.001). The largest proportion of patients was observed in the age groups 65 to 69 years and 70 to 74 years. For example, among patients aged 65 to 69 years, 3628 (43.31%) patients were alive or died from other causes, and 4748 (56.69%) patients died from prostate cancer. Among patients aged 80 to 84 years, 2676 (37.36%) patients were alive or died from other causes, and 4486 (62.64%) patients died from prostate cancer.

Race and ethnicity also differed significantly between the outcome groups (χ^2^ = 245.70, *p* < 0.001). Among non-Hispanic White patients, 2810 (43.52%) did not die from prostate cancer, whereas 3647 (56.48%) died from prostate cancer. Among non-Hispanic Black patients, 133 (35.95%) patients were alive or died from other causes, and 237 (64.05%) patients died from prostate cancer. Hispanic patients showed a higher proportion of patients classified as alive or dead from other causes, 114 (78.08%) patients, compared with prostate cancer deaths, 32 (21.92%) patients.

Median household income inflation was significantly associated with cancer-specific death status (χ^2^ = 339.79, *p* < 0.001). Patients residing in the low income level, defined as < $60 k, had 2613 (35.94%) patients alive or dead from other causes and 4658 (64.06%) patients who died from prostate cancer. In contrast, among patients residing in high income areas (≥$100 k), 5302 (45.34%) were either alive or died from causes other than prostate cancer, while 6392 (54.66%) died from prostate cancer.

Metastatic disease at diagnosis showed significant differences by outcome. Bone metastasis was present in 35,118 patients and was significantly associated with outcome (χ^2^ = 199.07, *p* < 0.001). Among patients with bone metastasis, 14,761 (42.03%) patients were alive or died from other causes, and 20,357 (57.97%) patients died from prostate cancer. Liver metastasis was present in 1703 patients and showed a large difference in outcome (χ^2^ = 193.60, *p* < 0.001). Among patients with liver metastasis, 402 (23.61%) patients were alive or died from other causes, and 1301 (76.39%) patients died from prostate cancer. Lung metastasis was also associated with outcome (χ^2^ = 4.62, *p* = 0.032). Brain metastasis was present in 425 patients and was significantly associated with outcome (χ^2^ = 15.33, *p* < 0.001).

Treatment variables also differed by outcome. Radiation therapy was recorded for 11,828 patients and showed a significant difference between groups (χ^2^ = 19.06, *p* < 0.001). Chemotherapy was administered in 6896 patients and was also associated with outcome (χ^2^ = 17.55, *p* < 0.001).

Lastly, survival time differed significantly between the groups (*t* = 73.76, *p* < 0.001). Patients who were alive or died from other causes had a mean survival time of 49.88 (41.17) months, whereas patients who died from prostate cancer had a mean survival time of 28.30 (26.81%) months.

Table 2 below presents multivariable Cox proportional hazards model for cancer-specific mortality in advanced prostate cancer.

After adjustment for demographic, socioeconomic, clinical, and treatment variables, several factors remained significantly associated with cancer-specific mortality.

Race and ethnicity were associated with survival. Compared with non-Hispanic White patients, non-Hispanic Black patients had a higher risk of prostate cancer-specific death (aHR = 1.15, 95% CI: 1.00 to 1.31, *p* = 0.046). Non-Hispanic Asian or Pacific Islander patients had a lower risk of death (aHR = 0.84, 95% CI: 0.79 to 0.89, *p* < 0.001). Non-Hispanic American Indian or Alaska Native patients had a higher risk of death (aHR = 1.15, 95% CI: 1.10 to 1.20, *p* < 0.001). Hispanic patients had a lower risk of death compared with non-Hispanic White patients (aHR = 0.32, 95% CI: 0.22 to 0.46, *p* < 0.001).

Age was also associated with mortality. Patients aged 80 to 84 years had a higher risk of death compared with those aged 40 to 44 years (aHR = 1.25, 95% CI: 1.04 to 1.51, *p* = 0.019). The risk was further increased among patients aged 85 to 89 years (aHR = 1.55, 95% CI: 1.29 to 1.87, *p* < 0.001) and among patients aged 90 years or older (aHR = 2.05, 95% CI: 1.69 to 2.48, *p* < 0.001).

Metastatic sites at diagnosis showed important differences. Liver metastasis was associated with a substantially higher risk of death (aHR = 2.15, 95% CI: 2.03 to 2.28, *p* < 0.001). Brain metastasis was also associated with increased risk (aHR = 1.60, 95% CI: 1.42 to 1.80, *p* < 0.001). Lung metastasis showed a modest increase in risk (aHR = 1.10, 95% CI: 1.05 to 1.15, *p* < 0.001). Bone metastasis was not significantly associated with mortality (aHR = 0.98, 95% CI: 0.96 to 1.01, *p* = 0.132).

Treatment variables showed mixed associations. Radiation therapy was not significantly associated with mortality (aHR = 0.98, 95% CI: 0.95 to 1.01, *p* = 0.126). Chemotherapy was associated with a higher risk of death (aHR = 1.11, 95% CI: 1.07 to 1.15, *p* < 0.001). However, this finding should be interpreted with caution, as it likely reflects confounding by indication. In clinical practice, chemotherapy is typically administered to patients with more advanced disease, higher tumor burden, or aggressive disease variants. Therefore, the observed association is more likely to reflect underlying disease severity rather than a direct effect of chemotherapy on mortality.

Median household income inflation showed a gradient in survival. Compared with patients residing in the low income level, those living in the upper-middle income level had a lower risk of death (aHR = 0.90, 95% CI: 0.87 to 0.94, *p* < 0.001). Patients residing in high income counties also had a lower risk of death (aHR = 0.83, 95% CI: 0.80 to 0.87, *p* < 0.001).

**Figure 1:**
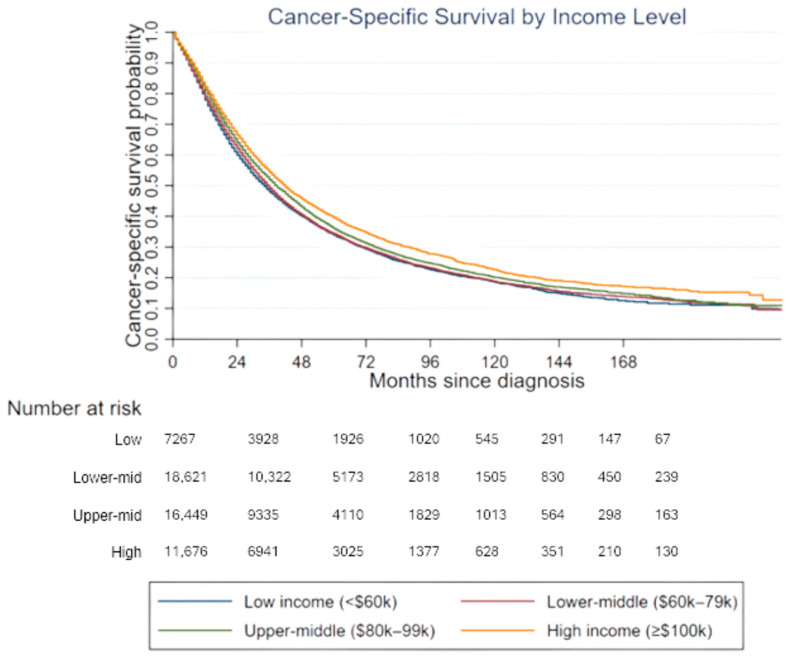
Cancer-specific survival by median household income inflation. Kaplan–Meier curves showing cancer-specific survival across income groups. The number of patients at risk at each time point is displayed below the x-axis. Differences between groups were evaluated using the log-rank test. Survival probability represents the proportion of patients who remained free from prostate cancer-specific death during follow up. The figure was generated by authors using Stata version 18 [22].

Figure 1 illustrates cancer-specific survival according to median household income inflation.

The survival curves show differences in cancer-specific survival across income groups over time. Patients residing in the high income level demonstrated higher survival probabilities throughout follow up compared with patients residing in the lower income level. Patients in the low income and lower-middle income groups showed lower survival probabilities. This pattern was observed during both the early and later periods of follow up. The separation between survival curves became more apparent after approximately two years of follow up and remained visible through the later months of observation.

## 4. Discussion

This study examined racial and socioeconomic disparities in stage at diagnosis and cancer-specific survival among patients with advanced prostate cancer using a large population-based dataset. Several important patterns were observed. Differences in cancer-specific death were identified across racial and ethnic groups, with higher mortality observed among non-Hispanic Black patients and non-Hispanic American Indian or Alaska Native patients, while Hispanic and non-Hispanic Asian or Pacific Islander patients showed lower mortality compared with non-Hispanic White patients. Age was also associated with survival, with higher mortality observed among older patients, particularly those aged 80 years and above. Metastatic disease at diagnosis showed substantial differences, especially for liver and brain metastases, which were associated with higher mortality risk. Socioeconomic status, represented by median household income, demonstrated a gradient in survival, where patients in higher income groups had lower mortality compared with those in lower income groups [22]. Prior population-based analyses have similarly demonstrated significant racial and ethnic differences in cause-specific mortality among patients with prostate cancer over extended follow-up periods, further supporting the role of systemic factors in shaping survival outcomes [23]. These findings suggest that structural determinants of health contribute to persistent survival disparities in metastatic prostate cancer. Factors such as differential access to early detection, timely systemic therapy, and high-volume oncology centers may play an important role in these observed differences [7,11].

Hispanic patients demonstrated a lower adjusted risk of prostate cancer-specific mortality in this analysis. However, this finding should be interpreted with caution given the relatively small sample size (*n* = 146) and the limitations of the SEER dataset. The registry does not capture important factors such as immigration status, nativity, comorbid conditions, or individual-level socioeconomic characteristics, which may influence survival outcomes. In addition, the Hispanic population is heterogeneous, comprising diverse subgroups with varying health profiles, cancer risks, and outcomes [24,25,26]. Therefore, this observed association should be considered descriptive and hypothesis generating rather than indicative of an underlying causal mechanism.

Prior research has reported persistent disparities in prostate cancer outcomes across racial groups. Studies have shown that Black men experience higher mortality from prostate cancer despite advances in diagnosis and treatment [8]. Reviews of racial disparities suggest that these differences reflect a complex interaction between social determinants, health care access, and biological variation rather than a single explanatory factor [10,17,27,28]. Socioeconomic status also plays an important role in prostate cancer outcomes. Lower income populations often experience delays in diagnosis, reduced access to specialized care, and barriers to treatment initiation, which may contribute to worse outcomes [11,13,29,30]. These findings are consistent with prior population-based studies demonstrating that socioeconomic disadvantage is associated with later stage at diagnosis and poorer survival outcomes in prostate cancer [10,16,23]. This relationship likely reflects differences in access to health care services, early detection, and timely treatment, which contribute to disparities in outcomes across population groups. These observations are consistent with the findings of this study, where lower income groups showed higher proportions of prostate cancer death compared with patients living in higher income areas. Disparities in metastatic disease at diagnosis have also been documented. Previous studies have reported that patients with limited access to health care services are more likely to present with advanced or metastatic disease, which is associated with poorer survival outcomes [4,19].

Current clinical recommendations in the United States emphasize early detection and risk-based management of prostate cancer. Screening strategies involving prostate-specific antigen testing and shared decision-making are intended to identify disease at earlier stages and reduce mortality risk [1]. Management recommendations also highlight individualized treatment strategies based on disease stage, patient age, and comorbid conditions [31]. Options such as active surveillance are recommended for selected patients with lower-risk disease to reduce unnecessary treatment while maintaining careful monitoring [2]. The presence of metastatic disease requires more intensive treatment strategies, including systemic therapy and other targeted interventions. These recommendations emphasize the importance of early detection and timely access to appropriate care, factors that may influence the disparities observed in this study. Variations in access to screening, diagnostic services, and treatment pathways may contribute to differences in stage at diagnosis and survival outcomes among different racial and socioeconomic groups [11,18,32].

Several clinical and biological mechanisms may contribute to the associations observed in this analysis. Differences in metastatic patterns may reflect variations in tumor biology, disease aggressiveness, and the timing of diagnosis. Metastatic spread to organs such as the liver or brain is often associated with more aggressive disease behavior and reduced survival [4]. Older age may also influence outcomes through the presence of comorbid conditions, reduced tolerance to intensive treatment, or delayed diagnosis. Socioeconomic differences may influence health-seeking behavior, access to specialized oncology care, and continuity of treatment [23]. Interestingly, patients aged 70–79 years did not demonstrate a significantly higher risk of prostate cancer-specific mortality compared with the youngest reference group. This finding has not been consistently reported in prior studies and may reflect the complex relationship between age and prostate cancer outcomes. Older patients are more likely to experience competing risks of death from non-cancer causes, which may attenuate observed cancer-specific mortality differences. In addition, treatment patterns may differ by age, with potential selection of healthier older individuals for therapy. Therefore, this observation should be interpreted cautiously. Racial differences in survival may also reflect variation in tumor characteristics, environmental exposures, and structural factors within health systems that affect care delivery [7,17]. These mechanisms may interact with each other and contribute to the differences in survival patterns observed across population groups.

## 5. Strengths and Limitations of This Study

This study has several strengths. The analysis used a large population-based dataset that captures diverse demographic and clinical characteristics across the United States. The large sample size allowed examination of multiple racial and socioeconomic groups and provided sufficient statistical power to evaluate survival differences. The use of cancer-specific survival also allowed a focused evaluation of prostate cancer-related mortality. Several limitations should also be considered. The analysis relied on registry-based data and did not include detailed clinical variables such as comorbid conditions, treatment adherence, or lifestyle factors that may influence survival outcomes. Some variables related to metastatic disease had missing values, which may introduce uncertainty in the estimates. Socioeconomic status was measured using area-level income rather than individual income, which may not fully capture personal socioeconomic conditions. The observational design of the dataset also limits the ability to assess causal relationships. In addition, the SEER database does not capture important clinical variables such as baseline comorbidities, performance status (e.g., ECOG), smoking history, or detailed disease burden metrics. These factors play a critical role in treatment selection and survival outcomes in advanced prostate cancer. The absence of these variables introduces the potential for residual confounding, particularly in analyses evaluating treatment effects and survival differences across population groups. Future research should explore additional clinical and social variables, including treatment patterns, health care access, and biological markers, to better understand the mechanisms underlying disparities in advanced prostate cancer outcomes [30].

## 6. Conclusions

This study demonstrates persistent disparities in cancer-specific survival among patients with advanced prostate cancer across racial and socioeconomic groups in the United States, consistent with prior population-based evidence. Patients from lower income groups and certain racial populations experienced less favorable survival outcomes, while higher income groups showed comparatively improved survival. Variations in metastatic disease and age at diagnosis were also associated with differences in survival, reflecting the complex interplay between disease characteristics, access to care, and social determinants of health. These findings reinforce existing evidence that inequities in health care access, early detection, and treatment delivery contribute to differences in prostate cancer outcomes. Addressing these disparities will require targeted strategies to improve equitable access to screening, timely diagnosis, and high-quality oncology care. Future research should focus on integrating clinical, biological, and health system factors to better understand and reduce disparities in survival across population groups.

## Figures and Tables

**Table 1:** Baseline characteristics of patients with advanced prostate cancer by cancer-specific death status (*n* = 54,062).

Characteristic	Alive/Died from Other Cause (*n* = 21,544)	Prostate Cancer Death (*n* = 32,518)	Test Statistic	*p*-Value
**Age group, *n* (%)**			χ^2^ = 241.34	<0.001
40–44 years	45 (27.78%)	117 (72.22%)	–	–
45–49 years	243 (32.27%)	510 (67.73%)	–	–
50–54 years	761 (35.18%)	1402 (64.82%)	–	–
55–59 years	1799 (39.40%)	2767 (60.60%)	–	–
60–64 years	2968 (41.64%)	4159 (58.36%)	–	–
65–69 years	3628 (43.31%)	4748 (56.69%)	–	–
70–74 years	3471 (42.91%)	4618 (57.09%)	–	–
75–79 years	3169 (41.25%)	4513 (58.75%)	–	–
80–84 years	2676 (37.36%)	4486 (62.64%)	–	–
85–89 years	1917 (35.56%)	3474 (64.44%)	–	–
≥90 years	867 (33.46%)	1724 (66.54%)	–	–
**Race/Ethnicity, *n* (%)**			χ^2^ = 245.70	<0.001
Non-Hispanic White	2810 (43.52%)	3647 (56.48%)	–	–
Non-Hispanic Black	133 (35.95%)	237 (64.05%)	–	–
Non-Hispanic Asian/Pacific Islander	1604 (47.85%)	1748 (52.15%)	–	–
Non-Hispanic American Indian/Alaska Native	3386 (38.71%)	5361 (61.29%)	–	–
Hispanic (All Races)	114 (78.08%)	32 (21.92%)	–	–
Non-Hispanic Unknown	13,497 (38.57%)	21,493 (61.43%)	–	–
**Median household income inflation, *n* (%)**	–	–	χ^2^ = 339.79	<0.001
Low income (<$60 k)	2613 (35.94%)	4658 (64.06%)	–	–
Lower-middle ($60–79 k)	6711 (36.02%)	11,921 (63.98%)	–	–
Upper-middle ($80–99 k)	6918 (42.02%)	9547 (57.98%)	–	–
High income (≥$100 k)	5302 (45.34%)	6392 (54.66%)	–	–
**Bone metastasis, *n* (%)**	–	–	χ^2^ = 199.07	<0.001
No	6783 (35.81%)	12,161 (64.19%)	–	–
Yes	14,761 (42.03%)	20,357 (57.97%)	–	–
**Liver metastasis, *n* (%)**	–	–	χ^2^ = 193.60	<0.001
No	21,142 (40.38%)	31,217 (59.62%)	–	–
Yes	402 (23.61%)	1301 (76.39%)	–	–
**Lung metastasis, *n* (%)**	–	–	χ^2^ = 4.62	0.032
No	20,236 (39.97%)	30,394 (60.03%)	–	–
Yes	1308 (38.11%)	2124 (61.89%)	–	–
**Brain metastasis, *n* (%)**	–	–	χ^2^ = 15.33	<0.001
No	21,414 (39.92%)	32,223 (60.08%)	–	–
Yes	130 (30.59%)	295 (69.41%)	–	–
**Radiation therapy, *n* (%)**	–	–	χ^2^ = 19.06	<0.001
No	15,915 (39.14%)	24,750 (60.86%)	–	–
Yes	4893 (41.37%)	6935 (58.63%)	–	–
**Chemotherapy, *n* (%)**	–	–	χ^2^ = 17.55	<0.001
No	18,955 (40.19%)	28,211 (59.81%)	–	–
Yes	2589 (37.54%)	4307 (62.46%)	–	–
Survival months, mean (SD)	49.88 (41.17)	28.30 (26.81)	t = 73.76	<0.001

*Values are presented as n (percent) for categorical variables and mean (SD) for continuous variables. Group comparisons were performed using chi-square tests for categorical variables and independent sample t tests for continuous variables. Cancer death indicates death attributable to prostate cancer. Metastatic variables represent presence of metastasis at diagnosis. SD indicates standard deviation.* Percentages are row percentages within each category. The table was generated by authors using Stata version 18 [22].

**Table 2:** Multivariable Cox proportional hazards model for cancer-specific mortality in advanced prostate cancer.

Variable	Adjusted HR	95% CI	*p*-Value
**Race/Ethnicity**	–	–	–
Non-Hispanic Black vs. Non-Hispanic White	1.15	1.00–1.31	0.046
Non-Hispanic Asian/Pacific Islander vs. Non-Hispanic White	0.84	0.79–0.89	<0.001
Non-Hispanic American Indian/Alaska Native vs. Non-Hispanic White	1.15	1.10–1.20	<0.001
Hispanic (All Races) vs. Non-Hispanic White	0.32	0.22–0.46	<0.001
Non-Hispanic Unknown vs. Non-Hispanic White	1.07	1.03–1.11	<0.001
**Age group at diagnosis**			
45–49 years vs. 40–44 years	0.86	0.70–1.06	0.150
50–54 years vs. 40–44 years	0.84	0.70–1.02	0.079
55–59 years vs. 40–44 years	0.80	0.66–0.96	0.018
60–64 years vs. 40–44 years	0.80	0.67–0.97	0.020
65–69 years vs. 40–44 years	0.80	0.67–0.97	0.020
70–74 years vs. 40–44 years	0.87	0.72–1.05	0.139
75–79 years vs. 40–44 years	0.99	0.82–1.19	0.905
80–84 years vs. 40–44 years	1.25	1.04–1.51	0.019
85–89 years vs. 40–44 years	1.55	1.29–1.87	<0.001
≥90 years vs. 40–44 years	2.05	1.69–2.48	<0.001
Bone metastasis (Yes vs. No)	0.98	0.96–1.01	0.132
Liver metastasis (Yes vs. No)	2.15	2.03–2.28	<0.001
Lung metastasis (Yes vs. No)	1.10	1.05–1.15	<0.001
Brain metastasis (Yes vs. No)	1.60	1.42–1.80	<0.001
Radiation therapy (Yes vs. No)	0.98	0.95–1.01	0.126
Chemotherapy (Yes vs. No)	1.11	1.07–1.15	<0.001
**Median household income inflation**	–	–	–
Lower-middle ($60–79 k) vs. Low income (<$60 k)	0.97	0.94–1.01	0.116
Upper-middle ($80–99 k) vs. Low income (<$60 k)	0.90	0.87–0.94	<0.001
High income (≥$100 k) vs. Low income (<$60 k)	0.83	0.80–0.87	<0.001

*Adjusted hazard ratios were estimated using a Cox proportional hazards regression model. The model included race and ethnicity, age group, metastatic sites at diagnosis, treatment variables, and median household income inflation. Hazard ratios represent the relative risk of prostate cancer-specific death. CI indicates confidence interval.* The table was generated by authors using Stata version 18 [22].

## Data Availability

Data used in this study were obtained from the SEER program maintained by the National Cancer Institute. The dataset is publicly available at https://seer.cancer.gov/ (accessed on 15 February 2026). upon completion of the required data-use agreement.
